# Walking the path of treatable traits in interstitial lung diseases

**DOI:** 10.1186/s12931-023-02554-8

**Published:** 2023-10-24

**Authors:** Francesco Amati, Paolo Spagnolo, Christopher J. Ryerson, Justin M. Oldham, Andrea Gramegna, Anna Stainer, Marco Mantero, Nicola Sverzellati, Donato Lacedonia, Luca Richeldi, Francesco Blasi, Stefano Aliberti

**Affiliations:** 1 Department of Biomedical Sciences, Humanitas University, Via Rita Levi Montalcini 4, 20072 Pieve Emanuele, Milan Italy; 2 IRCCS Humanitas Research Hospital, Respiratory Unit, Via Manzoni 56, 20089 Rozzano, Milan Italy; 3 Respiratory Disease Unit, Department of Cardiac, Thoracic, Vascular Sciences and Public Health, University of Padova, Padua, Italy; 4 Department of Medicine, University of British Columbia and Centre for Heart Lung Innovation, St. Paul’s Hospital, Vancouver, Canada; 5 Division of Pulmonary and Critical Care Medicine, Department of Internal Medicine, University of Michigan, Ann Arbor, MI USA; 6 Fondazione IRCCS Ca’ Granda Ospedale Maggiore Policlinico, Respiratory Unit and Cystic Fibrosis Adult Center, Milan, Italy; 7 Department of Pathophysiology and Transplantation, Università degli Studi di Milano, Milan, Italy; 8 Unit of Scienze Radiologiche, Department of Medicine and Surgery, University of Parma, Parma, Italy; 9 Department of Medical and Occupational Sciences, Institute of Respiratory Disease, Università degli Studi di Foggia, Foggia, Italy; 10 Fondazione Policlinico A. Gemelli IRCCS, Università Cattolica del Sacro Cuore, Rome, Italy

**Keywords:** Treatable traits, Biomarkers, Endotype, Phenotype, Interstitial lung diseases, Personalized medicine, Artificial intelligence

## Abstract

Interstitial lung diseases (ILDs) are complex and heterogeneous diseases. The use of traditional diagnostic classification in ILD can lead to suboptimal management, which is worsened by not considering the molecular pathways, biological complexity, and disease phenotypes. The identification of specific “treatable traits” in ILDs, which are clinically relevant and modifiable disease characteristics, may improve patient’s outcomes. Treatable traits in ILDs may be classified into four different domains (pulmonary, aetiological, comorbidities, and lifestyle), which will facilitate identification of related assessment tools, treatment options, and expected benefits. A multidisciplinary care team model is a potential way to implement a “treatable traits” strategy into clinical practice with the aim of improving patients’ outcomes. Multidisciplinary models of care, international registries, and the use of artificial intelligence may facilitate the implementation of the “treatable traits” approach into clinical practice. Prospective studies are needed to test potential therapies for a variety of treatable traits to further advance care of patients with ILD.

## Introduction

ILDs include more than 200 entities of either known or unknown etiology [1]. The “Oslerian paradigm” has represented with unquestionable merit the traditional approach to ILDs management in the last decades. This paradigm classifies the diseases by linking the principal organ system in which symptoms and signs manifest with anatomic and histopathology findings [2–4]. Traditionally, diagnostic classification of diseases is given based on a set of clinical features, and patients are treated accordingly [5, 6]. According to the “Oslerian paradigm”, the traditional goal of ILD management has been to determine the right treatment according to the initial and accurate diagnosis. However, this approach has become somewhat outdated with advances in technology allowing recognition of disease endotypes and phenotypes that can be treated with targeted interventions. Moreover, recent data have highlighted variability in disease pathogenesis across ILD subtypes resulting in unpredictable natural history and heterogeneous treatment response [7–10]. This evidence points towards the existence of “treatable traits”, specific disease characteristics that are clinically relevant and modifiable through pharmacological or non-pharmacological interventions [11]. “Treatable traits” approaches have been successfully implemented over the past decade in other chronic respiratory diseases, including bronchiectasis, asthma, and chronic obstructive pulmonary disease (COPD) [12, 13]. A number of treatable traits have also been identified in ILDs, such as progressive fibrosis or inflammation, either eosinophilic or neutrophilic [14–17]. The majority of these are phenotype-driven, while several studies are underway to stratify ILD patients according to clinically relevant endotypes [15–17]. Moving from a “disease-centered” to a “personalized” management approach that is based on specific treatable traits is a priority for the field, with the aim of identifying new targets to customize treatment and improve patients’ outcomes [8]. In this perspective, we describe the future potential of using treatable traits in the management of ILDs.

### The “splitting” approach in ILDs

**Fig. 1:**
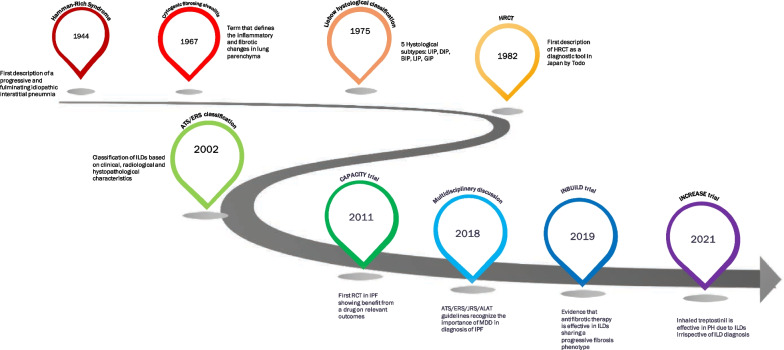
Milestones in history of ILD

ILDs are a heterogeneous group of conditions with overlapping clinical features, radiological and histological findings, as well as pathobiological underpinnings. This heterogeneity represents a substantial barrier in understanding disease mechanisms and developing efficacious and personalized treatments. The diagnosis and treatment of ILD has challenged physicians since the middle of the last century [18]. The first milestone in ILD diagnosis was the clinical-radiological-pathologic description by Louis Hamman and Arnold Rich in 1944 of four patients who died of progressive respiratory failure within 6 months of symptoms’ onset at the Johns Hopkins Hospital in the United States [18] (Fig. 1).

The term Hamman-Rich syndrome became a synonym for an acute interstitial pneumonia of unknown cause that rapidly progressed to pulmonary fibrosis and almost invariably resulted in death. In 1957, Rubin and Lubliner reviewed 48 cases of the Hamman-Rich syndrome and added 15 cases of their own [19]. It soon became evident that the course of this new entity was not always acute, progressive, or fatal. In 1964, thanks to Sheridan and colleagues, the concept of idiopathic interstitial pneumonia evolved from the acute (or subacute) and fulminant disease described by Hamman and Rich [20]. Subsequently, at the Brompton Chest Hospital in London, Scadding and Hinson coined the term “cryptogenic fibrosing alveolitis” to describe the inflammatory and fibrotic changes that occurred in the lung parenchyma of patients with pulmonary fibrosis of unknown origin.

Open lung biopsy rapidly emerged as the gold standard for diagnosing ILDs [21]. In 1975, according to his own clinical and pathologic data, Averill Liebow classified interstitial pneumonitis into five different histologic categories: usual interstitial pneumonitis (UIP), desquamative interstitial pneumonia (DIP), bronchiolitis obliterans interstitial pneumonia (BIP), lymphoid interstitial pneumonia (LIP), and giant cell interstitial pneumonia (GIP) [22]. In 1982, a key step towards the diagnosis and classification of ILDs was the implementation in clinical practice of the high-resolution computed tomography (HRCT) of the chest by Todo and coworkers [23], which substantially improved the ability to classify ILD subtypes through noninvasive means. Consequently, clinical, radiological, and, when available, histopathological data were integrated to improve diagnostic accuracy and implement classification of ILDs through the so-called “splitting” approach [24]. This approach allowed the design and conduct of randomized controlled trials (RCT)s for common ILDs, namely idiopathic pulmonary fibrosis (IPF) and systemic sclerosis-associated (SSc) ILD [25–29]. While this approach has resulted in the approval of several therapies to slow IPF and SSc-ILD, it left many other ILDs understudied, and it is limited by several factors. Firstly, ILD patients sharing the same diagnosis might respond differently to the same pharmacological treatment. Irrespective of ILD diagnosis, individuals with reduced telomere length endotype and/or known telomere-related mutations have more rapid disease progression and shorter lung transplant-free survival [30]. IPF patients who carry a specific polymorphism within the Toll-interacting protein (TOLLIP) gene might benefit from *N*-acetylcysteine (NAC), despite being ineffective for patients lacking this polymorphism and when studied in all-comers with IPF [31]. A European, multicenter, retrospective study demonstrated that IPF patients carrying mutations within Telomerase Reverse Transcriptase (TERT) or Telomerase RNA Component (TERC) gene may not benefit from pirfenidone in terms of reduction of lung function decline, although this drug is recommended for IPF [32]. Moreover, in a post-hoc analysis of two trials, IPF patients carrying rare variants within one telomere-related gene (TRG) showed a more rapid decline in forced vital capacity (FVC) than non-carriers [33]. Finally, a strong correlation has been reported between the presence of reduced telomere and the harmful effect of immunosuppressive medication in ILD patients [34]. Taken together, these findings suggest a more nuanced approach may be needed to effectively treat diverse ILDs.

Secondly, multiple comorbidities are common in ILD and have a detrimental effect on survival, especially if untreated [35–37]. Although clinicians should have a low threshold for suspecting comorbidities in patients with ILD, recent data show that treatment of relevant comorbidities is suboptimal, and often lacking altogether [35, 36].

Thirdly, data from both registries and multicenter studies show low-quality standards with regards to the management of ILD, including adherence to pharmacological treatment and referral to lung transplant centers, when indicated [37, 38].

### A “treatable traits” strategy: lessons learned from other chronic respiratory diseases

Chronic respiratory diseases are often complex and heterogeneous conditions that require individualized assessment and treatment. Precision medicine is defined as “treatments targeting the needs of individual patients on the basis of genetic, biomarker, phenotypic, or psychosocial characteristics that distinguish a given patient from others with similar clinical presentations” [39, 40]. The precision medicine strategy relies on the systematic evaluation of “treatable traits”, as originally reported for chronic airway diseases by Agusti et al. in 2015 [12]. According to this approach, patients are individually assessed for a specific set of treatable problems. The identification of treatable traits has led to the adoption of different and specific therapeutic strategies, thus going beyond the “Oslerian paradigm”. In the field of airway diseases, the Oslerian diagnostic classification might lead to sub-optimal management because specific molecular pathways and disease phenotypes are not taken into account [3, 4, 41]. Indeed, phenotypic and endotypic features of chronic airway diseases (e.g.: treatable traits) are variable, show non-linear dynamic interactions, and differentially regulate patterns and burden of the disease as well as response to treatment [42, 43]. Different trials using a “treatable traits” strategy have been conducted both in asthma and COPD [44, 45]. These studies have shown that a “holistic” approach based on the identification of treatable traits might improve outcomes if compared to a “guideline-based” approach. This represents an important step forward in the management of chronic airway diseases [44, 45].

### A “treatable traits” strategy in ILDs: integrating “lumping” and “splitting” approaches

We recently propose and summarize a “treatable traits” strategy for patients with ILDs [14] (Table 1). Treatable traits have been classified into four different domains (pulmonary, etiological, comorbidities, and lifestyle) along with the identification of related assessment tools, potential treatment options, and expected benefits.

**Table 1:** Treatable traits in interstitial lung diseases according to pulmonary, aetiological, comorbidities and lifestyle domains

Treatable trait	Assessment tool	(Potential) treatment option	Expected benefits of treatment*
*Aetiological*			
CTDs/Vasculitis	Clinical features Serum antibodies	Refer to rheumatologist Screening for extra-respiratory involvement Immunosuppressive drugs	Prevent or reduce lung damage Reduce mortality
Drugs	Drug history	Assess risk–benefit of stopping potentially harmful drug	Reduce lung damage Improve lung function
Exposure-related (organic and inorganic)	Environmental/ work/ domestic history of exposure Serum precipitins	Prevent or stop exposure	Prevent or reduce lung damage Improve outcomes Reduce mortality
Genetic	Family history Age of onset DNA genetic testing	Refer to geneticist Family screening Targeted therapy	Improve outcomes
*Lifestyle*			
Smoking	Patient reported Urine cotinine levels	Tobacco cessation support Nicotine replacement Antidepressant drug	Improve quality of life Improve lung function Prevent or reduce lung damage
Adherence to treatment	Patient and relatives feedback	Education Written instructions Self-management Family and social support	Improve outcomes
Exposure to air pollution	PM10 and NO2 concentrations	Reduce exposure	Reduce disease progression Reduce exacerbation
Lack of exercise/ Deconditioning of skeletal muscle	Cardiopulmonary exercise testing 6MWT	Prescribed exercise programs Pulmonary rehabilitation	Improve quality of life Improve lung function Improve exercise capacity
Diet	Patient reported	Diet instructions	Improve quality of life
*Pulmonary*			
Progressive fibrosis	Patient reported symptoms Pulmonary function tests HRCT	Optimization of therapy Consider antifibrotics Referral to lung transplant center	Slow lung function decline Reduce mortality Prevent exacerbation
Eosinophilic inflammation	HRCT BAL CBC	Steroid therapy Adjust immunosuppression	Prevent or reduce lung damage Improve quality of life
Neutrophilic inflammation	HRCT BAL CBC	Azithromycin	Reduce lung damage Improve quality of life
Acute exacerbation	HRCT BAL	Antifibrotic therapy Systemic glucocorticoids	Improve survival
Acute infection	Patient reported symptoms Sputum cultures BAL	Airway clearance Antibiotic therapy Prophylaxis with influenza and pneumococcal vaccination Adjust immunosuppression	Prevent exacerbation Reduce mortality Reduce hospitalization
Chronic infection or recurrent infection	Patient reported symptoms Sputum cultures BAL	Airway clearance Adjust immunosuppression Consider prophylactic antibiotics	Prevent exacerbation Improve quality of life Slow lung function decline
Chronic respiratory failure	Patient reported symptoms ABG 6MWT Polysomnography	Long term oxygen therapy Non-invasive ventilation Referral to lung transplant center Pulmonary rehabilitation Palliative care	Improve quality of life Improve survival
Intractable chronic cough	Patient reported symptoms Scores (LCQ, VAS, CQLQ)	Antitussive Thalidomide Gabapentin	Improve quality of life
Emphysema / Obstructive ventilatory defects	Pulmonary function tests HRCT	Bronchodilator therapy	Improve quality of life Slow lung function decline
*Comorbidities*			
GERD	Symptoms Oesophageal pH monitoring Manometry Upper Endoscopy	Diet instructions PPIs, H2-receptor antagonists, pro-kinetics Fundoplication	Improve outcomes Reduce lung damage
Pulmonary hypertension	Echocardiography Consider RHC	Referral to lung transplant center Trial with PH targeted therapies in selected patients (e.g.: Treprostinil) Oxygen/Non-invasive ventilation	Improve quality of life Improve outcomes Reduce mortality
Congestive heart failure	NT-proBNP Echocardiography	Targeted pharmacological treatment ICD implantation	Improve quality of life Reduce mortality
OSA	Sleep study	Diet instructions CPAP treatment	Improve quality of life Reduce mortality
Lung cancer	HRCT PET- CT Biopsy	Surgical resection Radiotherapy Chemotherapy	Reduce mortality
Diabetes	Fasting glucose persistently above 125 mg/dl Random glucose levels above 200 mg/dl occurring in the context of high-dose glucocorticoid therapy	Diet instructions Lifestyle instructions Insulin Oral hypoglycemics	Improve quality of life Reduce systemic complication of diabetes Reduce mortality
Osteoporosis/Osteopenia	Bone densitometry	Diet instructions Lifestyle instructions Pharmacological therapy	Improve quality of life Reduce risk of fractures Reduce mortality
Pulmonary embolism	CT pulmonary angiogram	Anticoagulants	Reduce mortality
Obesity	BMI	Diet instructions Pulmonary rehabilitation Bariatric surgery	Improve quality of life Improve exercise tolerance
Cachexia/Malnutrition	BMI	Nutritional support Pulmonary rehabilitation	Improve quality of life
Frailty	FFP binary score Frailty Index	Nutritional supportive Pulmonary rehabilitation Physical activity programs	Improve quality of life Reduce mortality
Anxiety/Depression	Patient reported scores (K-BILD; SGRQ-IPF)	Counseling/cognitive behavioural therapy Antidepressant/Anxiolytics Pulmonary rehabilitation	Improve quality of life Improve adherence to treatment

*CTD* connective tissue disease, *PM10* particulate matter 10, *NO2* nitrogen dioxide, *6MWT* six-minute walk test, *HR-CT* high-resolution computed tomography, *BAL* bronchoalveolar lavage, *CBC* complete blood count, *ABG* arterial blood gas, *LCQ* leicester cough questionnaire, *VAS* visual analogue scale, *CQLQ* cough-specific quality of life questionnaire, *GERD* gastro-esophageal reflux disease, *PPI* proton pump inhibitor, *RHC* right heart catheterization, *NT-proBNP* N-terminal pro-B-type natriuretic peptide, *ICD* implantable cardioverter defibrillator, *OSA* obstructive sleep apnea, *CPAP* continuous positive airway pressure, *PET-CT* Positron emission tomography computed tomography, *BMI* body mass index, *FFP* Fried’s frailty phenotype, *K-BILD* King's brief interstitial lung disease, *SGRQ-IPF* St George's respiratory questionnaire idiopathic pulmonary fibrosis

^*^Most of benefits are speculative. Most of them are based on case report/case series or benefits coming from evidences in other diseases

From a pulmonary perspective, patients with a progressive pulmonary fibrosis (PPF) phenotype represents one of the most promising treatable traits [16, 46–50]. PPF refers to a spectrum of ILDs that share a phenotype characterized by an increasing extent of fibrosis on HRCT, decline in lung function, and worsening symptoms, resulting in decreased quality of life, and early death [51]. It has been hypothesized that ILDs with this phenotype may also share pathobiological mechanisms regardless of their underlying cause and thus may also respond to similar treatments. Indeed, in patients with PPF, nintedanib, an intracellular tyrosine kinase inhibitor with antifibrotic properties, reduces the rate of disease progression irrespective of the underlying ILD diagnosis [52–54]. Interestingly, molecular pathways targeted by nintedanib are augmented to a similar magnitude in lung tissue from several PPF, regardless of etiology [15, 54]. Pirfenidone, a compound with antifibrotic, anti-oxidant, and anti-inflammatory properties, might also represent an effective treatment for patients with PPF ILDs [55–57]. Taken together, these data suggest that the PPF phenotype of ILD is a clinically relevant and modifiable trait.

From an etiological perspective, the elimination of the inciting agent is an essential intervention in patients with exposure-related ILDs. A recent study showed that a standardized interview is able to reveal relevant inhalational exposures in most patients across all types of ILDs [38]. Exposures were markedly different based on demographics and were associated with worse transplant-free survival [38]. A thorough professional cleaning of the domestic or working environment needs to be undertaken if the patient remains within that environment [58, 59].

From a comorbidity perspective, the majority of ILD patients share additional complexity related to aging, concomitant conditions and extra-respiratory involvement [60–62]. These traits are often underestimated, leading to a vicious cycle that results in an accelerated clinical deterioration and worse prognosis [17, 60, 61, 63, 64]. A specific trait might be a major determinant of disease progression and may further influence other disease traits. This is the case of cachexia, frailty, and pulmonary hypertension (PH) among others. As an example, ILD patients frequently suffer from cachexia that often correlates with rapid clinical deterioration and early death [65–67]. Targeting cachexia in patients considered for lung transplant could have a substantial impact not only on body weight, but also on exercise tolerance, skeletal muscle strength, anxiety, and depression, thus potentially impacting the final decision on whether to proceed with transplantation [66, 68, 69]. Similarly, frailty is common in patients with ILDs and is strongly associated with dyspnea and clinical outcomes, including hospitalizations and mortality [69–71]. Frail patients generally have several comorbidities that are common causes of exclusion from clinical trials. In patients with ILD, physical frailty is an important determinant of prognosis and may represent a modifiable target for intervention [72, 73]. PH secondary to ILDs is associated with worse outcomes such as dyspnea, quality of life, and short-term mortality [74]. No therapies are currently approved for PH in ILD [74, 75]. However, inhaled treprostinil has been shown to improve exercise capacity, as assessed by 6-min walk test, compared with placebo in patients with PH due to ILD [76].

From a lifestyle/behavioral perspective, there are multiple studies showing the association of ILDs with air pollution, although the mechanisms through which air pollution leads to the development and worsening of ILD remain speculative [77–81]. Air pollution, as demonstrated by the PM_10_ concentration, is associated with an increasing rate of pulmonary function decline in IPF, as well as with higher risk of disease exacerbations [77, 81]. Thus, it is important to integrate markers of air pollution in the clinical evaluation of ILD patients irrespective of the underlying diagnosis.

### The multidisciplinary team as a tool to implement the “treatable trait” strategy in ILD

Multidisciplinary team (MDT) has become the diagnostic gold standard for ILD, particularly IPF [82–84]. The “treatable traits” approach requires the MDT be actively involved non only in disease diagnosis but also in the identification and management of pulmonary, aetiological, comorbidities, and lifestyle-related treatable traits with the aim of improving patients’ outcomes (Table 2) [82, 83]. This is the case, for example, of radiologists in the example of radiological progression of pulmonary fibrosis, cardiologists in the case of coexisting PH, or geriatricians for patients with frailty, or rheumatologist for possible connective tissue disease-ILD.

**Table 2:** Specialist competencies within the ILD multidisciplinary care team

Specialist	Competencies	Treatable trait areas of focus
Chest physician (coordination of the multidisciplinary team)	✓ Diagnosis confirmation and investigation of etiology ✓ Performance and evaluation of PFTs ✓ Bronchoscopy ✓ Medication review (side effects of treatment and polypharmacy review) ✓ Ensure patient’s adherence to treatment ✓ Management of respiratory failure, LTOT, NIV ✓ Identification of patients who are candidates for lung transplant ✓ Identification and management of acute exacerbations ✓ Identification of patients eligible for RCTs ✓ Monitoring of disease severity and potential complications ✓ End-of-life care	✓ Etiological ✓ Lifestyle ✓ Pulmonary ✓ Comorbidities
Rheumatologist	✓ CTD suspicion and diagnosis ✓ Identification of extra-respiratory involvement ✓ Co-management and prescription of immunosuppressive and antifibrotic drugs	✓ Etiological ✓ Comorbidities
Physiotherapist and/or respiratory therapist	✓ Airway clearance ✓ Pulmonary rehabilitation ✓ Inhaled therapy management	✓ Lifestyle ✓ Pulmonary
Nurse	✓ Coordinate input of other healthcare professionals ✓ Support patients and their families to recognize symptoms so as to avoid complications ✓ Ensure patient’s adherence to treatment	✓ Lifestyle
Pathologist	✓ Support etiological diagnosis ✓ Identification of progressive fibrosis ✓ Identification of complications	✓ Etiological ✓ Comorbidities
Radiologist	✓ Support etiological diagnosis ✓ Identification of progressive fibrosis ✓ Identification of AE ✓ Identification of complications	✓ Etiological ✓ Comorbidities
Gastroenterologist	✓ Diagnosis and management of GERD ✓ Management of gastrointestinal side effects of treatment (e.g.: antifibrotics) ✓ Evaluation and management of extra-respiratory involvement	✓ Comorbidities
Geneticist	✓ Diagnosis of genetic disorders ✓ Genetic counseling for patient and relatives	✓ Lifestyle ✓ Etiological
Infectious disease specialist	✓ Advice on potential prophylaxis in patients treated with immunosuppressive drugs ✓ Treatment of acute and chronic infection	✓ Multisystem
Cardiologist	✓ Diagnosis and management of RHF ✓ Evaluate heart involvement in some ILDs (e.g.: sarcoidosis or scleroderma)	✓ Comorbidities
Psychologist	✓ Management of anxiety and depression ✓ Family support ✓ End-of-life care	✓ Lifestyle
Endocrinologist	✓ Diagnosis and management of osteoporosis and diabetes in patients treated with chronic steroid therapy	✓ Comorbidities
Nutritionist	✓ Diet instruction	✓ Lifestyle ✓ Comorbidities
Geriatrician	✓ Identification of frail patients ✓ Medication review ✓ Familial and social support	✓ Lifestyle ✓ Comorbidities
Pharmacologist	✓ Medication review (side effects of treatment and polypharmacy review) ✓ End-of-life care	✓ Pulmonary
Thoracic Surgeon	✓ Surgical biopsy ✓ Co -management of lung cancer ✓ Lung transplant	✓ Etiological ✓ Comorbidities
General practitioner	✓ Medication review (side effects of treatment and polypharmacy review) ✓ Ensure patient’s adherence to treatment ✓ Support patients and their families to recognize symptoms so as to avoid complications ✓ End-of-life care	✓ Lifestyle ✓ Pulmonary ✓ Comorbidities

*PFTs* pulmonary function tests, *LTOT* long-term oxygen therapy, *NIV* non-invasive ventilation, *RCTs* randomized controlled trials, *CTD* connective tissue disease, *ILD* interstitial lung disease, *GERD* gastro-esophageal reflux disease, *RHF* right heart failure

### “Treatable traits” strategy in ILD: challenges and opportunities

#### Definitions and prevalence of treatable traits

Data on definition and prevalence of treatable traits in ILD are limited and mostly derived from monocentric observational studies [15, 46–48, 63]. The heterogeneity in prevalence of each trait depends on the intensity and type of the diagnostic workup, the threshold used to describe the trait as clinically relevant, and the setting where the trait has been identified (e.g.: primary, secondary or tertiary-care). For example, the prevalence of gastro-esophageal reflux disease (GERD) as a comorbidity varies substantially depending on the methods used to ascertain its presence or absence (e.g., medical records versus sensitive oesophageal pH-monitoring) [63, 85, 86]. Accordingly, the prevalence of GERD in IPF ranges from 0 to 94% [63]. Multicentre, prospective studies as well as international registries shared across different settings and countries are needed to improve the accuracy of estimates for both prevalence and characteristics of each single treatable trait [87–89].

#### Lack of endotypes in ILDs and related biomarkers

Recent RCTs in asthma and COPD have successfully tested that a biomarker-guided therapy directed to a specific treatable trait is superior to a symptom-guided therapy [90–92]. Specific biomarkers are already used in clinical practice to identify several endotypes in chronic respiratory diseases (e.g.: blood eosinophilia and the T2-high endotype in patients with asthma) [90]. However, there are few reliable biomarkers available for use in ILD. Biomarker discovery and identification of endotypes in ILDs represent a growing field of research owing to their potential clinical relevance. The pathophysiology of ILD is multifactorial, involving a complex interaction between host and environmental factors that results in the activation of multiple overlapping profibrotic pathways [93]. As a result, IPF and other ILDs may be indistinguishable from each other and have similarly variable and unpredictable clinical course, despite different underlying etiologies [94–96]. In addition, IPF and non-IPF PF-ILD patients share similar rates of functional decline, disease progression, and response to treatment [54].

Thus identify, develop, and validate molecular endotypes in ILD through the use of specific biomarkers is of paramount importance in order to evaluate disease activity and guide decision-making. Molecular biomarkers identified so far in ILDs, along with their clinical implications, are summarized in Table 3. However, most of these have been investigated in observational and retrospective studies without assay validation or replication in an external cohort. Issues related to reproducibility and inter-individual variability has also been highlighted for circulating biomarkers [128]. Thus, baseline and longitudinal measurement of specific and sensitive biomarkers in large and prospectively collected cohorts is of paramount importance for improving disease management. Machine learning and artificial intelligence (AI) might offer the opportunity to identify combinatorial biomarkers, which, in turn, may be more informative than markers used in isolation [129, 130].

**Table 3:** Molecular biomarkers identified in ILDs that are associated with relevant outcomes

Biomarker	Matrix	Disease*	Field of action	Prognostic relevance	Potential treatment
Short telomere length [34, 30, 97–101]	Peripheral blood leucocytes	IPF HP Unclassifiable ILD IPAF CTD-ILD	Dysfunctional alveolar repair	Higher post-transplant morbidity Significantly increased risk of harm in patients receiving immunomodulatory treatment	Placenta derived mesenchymal stromal cells
TOLLIP gene variant [31, 102]	Peripheral blood leucocytes	IPF	Immune dysregulation	Lung function decline Mortality	NAC
MUC5B promoter polymorphism [97–99, 103]	Peripheral blood leucocytes	IPF	Dysfunctional alveolar repair	Mortality	Unknown
KL-6 [104–113]	Serum BAL	CTD-ILD NSIP PAP CHP IPF	Dysfunctional alveolar repair	Severity of disease Risk of progression Risk of exacerbation Radiological scores	Unknown
Surfactant protein D [104–109, 112, 113]	Serum	IPF	Dysfunctional alveolar repair	Risk of progression	Unknown
MMP-7 [105, 106, 110, 114]	Serum	IPF CHP	Extracellular matrix turnover and remodeling	Lung function decline Mortality	Unknown
YKL-40 [104, 115, 119, 120]	Serum BAL	IPF CHP CTD-ILD Sarcoidosis	Extracellular matrix turnover and remodeling	Risk of progression Risk of exacerbation Mortality	Unknown
CCL-18 [104, 111, 116]	Serum	IPF	Immune dysregulation	Lung function decline Mortality	Unknown
IL-6 [117, 118]	Serum	CTD-ILD	Immune dysregulation	Lung function decline Mortality	Tocilizumab
PRO-C3 and PRO-C6 [121, 122]	Serum	IPF	Extracellular matrix turnover and remodeling	Risk of progression	Unknown
CRPM [123]	Serum	IPF	Extracellular matrix turnover and remodeling	Lung function decline	Unknown
Periostin [124]	Serum	IPF CHP	Extracellular matrix turnover and remodeling	Lung function decline Mortality Risk of exacerbation	Unknown
VCAM-1 [125, 126]	Serum	CTD-ILD IPF Unclassifiable ILD CHP	Immune dysregulation	Lung function decline Mortality	Unknown
CXCL 13 [126, 127]	Serum	CTD-ILD CHP IPF Unclassifiable ILD	Immune dysregulation	Lung function decline Mortality	Unknown

*IPF* idiopathic pulmonary fibrosis, *HP* hypersensitivity pneumonitis, *IPAF* interstitial pneumonia with autoimmune features, *CTD-ILD* connective tissue disease-interstitial lung disease, *TOLLIP* toll interacting protein, *NAC* N-acetyl cysteine, *MUC5B* Mucin 5B, *KL-6* Krebs von den Lungen 6, *BAL* bronchoalveolar lavage, *NSIP* non-specific interstitial pneumonia, *PAP* pulmonary alveolar proteinosis, *MMP-7* matrix metallopreinase 7, *YKL-40* chitinase-3-like protein 1, *CCL-18* C–C Motif Chemokine Ligand 18, *IL-6* Interlukin-6, *CRPM* serum matrix metalloproteinase-degraded C-reactive protein, *VCAM-1* vascular cell adhesion protein 1, *CXCL 13* C-X-C motif chemokine 13

^*^Disease(s) in which the biomarker has been identified

#### Information technology and artificial intelligence

Information technology (IT) and AI are increasingly integrated in clinical practice [90]. Deep learning-based HRCT image algorithms has the potential to improve diagnostic accuracy and predict disease behavior in ILD [131–134]. Similarly, machine learning and genomic analysis have been employed for the categorization of ILD morphology in the setting of computational pathology [130, 135, 136]. Several other traits might be suitable targets of AI. In this context, one of the most interesting and promising traits is represented by air pollution and its role in the pathogenesis and progression of ILDs [77, 80, 81]. Although air pollution is quantifiable, predicting air quality is a complex task due to the dynamic nature, and high variability in space and time of pollutants and particulates. AI and deep learning algorithms might provide useful information on these dynamic changes. Thus, data-driven decision-making in ILDs can be leveraged by incorporating deep learning algorithms into clinical practice. However, large datasets for adequate training of deep learning algorithms are needed to make effective this approach. Open science research and collaboration between academia and industry should be encouraged to identify and standardize treatable traits through deep learning analysis and algorithms.

#### Managing treatable traits

The management of treatable traits in ILD remains a matter of debate, with no clear guidance for (1) the prioritization of treatable traits for management; (2) the timing of treatable trait intervention and (3) the integration of patient preferences and values into this approach. Evidence from other chronic pulmonary diseases has demonstrated improved outcomes when patients are included at all stages in the decision-making process [137].

#### How to identify and interpret treatable traits over time

ILD may evolve within weeks, months, or years. The evaluation of treatable traits should thus be assessed repeatedly throughout the disease course. Dynamic biomarker change may reflect an evolving treatable trait, prompting a change in management strategy. One example is the regression of ground glass opacities (a “radiological biomarker”) in the context of local inflammation (the “trait”) following steroid treatment, which might lead physicians to re-define their management strategy and address other disease traits. An additional example is the development of overt autoimmune disease in a patient initially diagnosed with “idiopathic” nonspecific interstitial pneumonia (NSIP) [138, 139]. For such patients, a regular clinical review of new treatable traits related to the autoimmune disease will be critical for the appropriate management of the extra-respiratory autoimmune manifestations.

#### How to make the “treatable traits” approach feasible across different healthcare systems

While a “treatable traits” approach in ILD may be feasible in high-income countries with well-resourced hospitals and strong multidisciplinary networks, this can be challenging in under-resourced countries. For example, the implementation of deep learning technologies within the MDT is strictly dependent on local resources and varies across countries [140]. However, although this approach is initially highly resource-consuming, it would likely become cost-saving over time. The presence of a multi-disciplinary discussion (MDD) can optimize patients’ flow and avoid unnecessary delays in pharmacological and non-pharmacological interventions [141]. Beyond establishment of an ILD diagnosis and initial management plan, additional multidisciplinary input from a variety of healthcare professionals can also have a prominent role in identifying and managing treatable traits. For example, the role of a nutritionist in the management of ILD patients with obesity can potentially have a positive impact not only on the lifestyle domain but also on other traits, such as GERD, obstructive sleep apnea (OSA), or chronic cough. However, several questions remain unanswered, especially with regard to the optimal structure of such a multidisciplinary care team and which patients would benefit most from such intervention.

#### How to implement a “treatable traits” approach into the current landscape of ILD research

The use of traditional disease diagnosis has resulted in a large proportion of patients with ILDs being excluded from RCTs [25, 142, 143]. Using an “Oslerian paradigm”, 10–20% of ILD patients remain “unclassifiable” despite an intensive diagnostic work-up [82, 144]. Patients with unclassifiable ILD have a heterogeneous clinical course and are generally excluded from RCTs, although a recent phase 2 trial has investigated the efficacy and safety of pirfenidone in this patient subset [56]. Moreover, the benefit of pharmacological and non-pharmacological treatments is frequently limited by their effect on a single disease pathway, partially explaining the modest impact of these treatments on clinically relevant outcomes [25, 142, 143]. Trials in COPD and asthma showed that the implementation of a “treatable traits” strategy improves patients’ outcomes [44, 45]. The continued identification and implementation of molecular signature-based approaches to treatable traits may overcome these relevant limitations [134, 135, 145].

## Conclusion

**Fig. 2:**
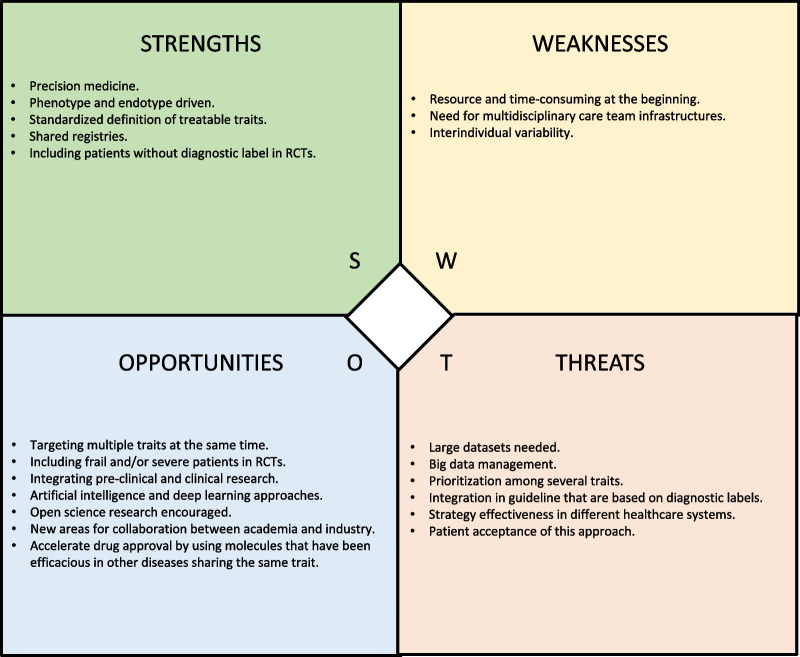
SWOT analysis of the treatable trait strategy in ILD. SWOT is an acronym that identifies the four elements of the analysis: strengths (S), weakness (W), opportunities (O), and threats (T). A SWOT analysis allows to critically explore advantages and disadvantages resulting from internal and external factors

ILDs comprise a group of complex and heterogeneous diseases that remain a challenge for treating physicians. The strategy described in this perspective focusses on key clinical treatable traits and underscores the importance of biomarkers for identifying clinically relevant treatable traits in patients with ILD, with the goal of using this approach in the daily management of ILD to improve outcomes. Compared to traditional ILD management, the treatable traits strategy is based on a “proactive” rather than “reactive” approach. This subtle distinction implies a relevant meaning that shift toward increasingly patient-centered medicine, moving beyond the “one size fits all” view. This is in line with an evolving healthcare system that is predictive, preventive, personalized and participatory, the so called “P4 medicine” [146, 147]. From a preventive point of view, Interstitial lung abnormalities (ILAs) are increasingly recognized on chest CTs [148]. Although the clinical relevance of ILA remains to be clarified, increased mortality as well as reduced pulmonary function have been reported [149, 150]. Thus, identification of biomarkers able to predict disease progression in ILA are of paramount importance. Finally, a SWOT analysis of the “treatable traits” proposal is provided in Fig. 2. Importantly, the identification and management of treatable traits should not be restricted to the pulmonary domain. In fact, the detection of extra-pulmonary comorbidities as well as lifestyle and behavioral traits, although not strictly biologically related to ILDs, might impact quality of life and clinical outcomes. Multidisciplinary models of care, international registries, and the use of AI may facilitate the implementation of the “treatable traits” approach into clinical practice. This perspective aims to foster collaboration among the respiratory community, regulatory agencies and pharmaceutical industries. The “treatable traits” approach is a clinical and research priority, with properly designed cohorts and RCTs needed to test potential therapies for a variety of treatable traits to further advance care of patients with ILD.

## Data Availability

Not applicable.

## Abbreviations

ILD: Interstitial lung disease
COPD: Chronic obstructive pulmonary disease
UIP: Usual interstitial pneumonitis
DIP: Desquamative interstitial pneumonia
BIP: Bronchiolitis obliterans interstitial pneumonia
LIP: Lymphoid interstitial pneumonia
GIP: Giant cell interstitial pneumonia
HRCT: High-resolution computed tomography
RCT: Randomized controlled trial
IPF: Idiopathic pulmonary fibrosis
SSc: Systemic sclerosis
TOLLIP: Toll-interacting protein
TERT: Telomerase reverse transcriptase
TERC: Telomerase RNA component
NAC: *N*-Acetylcysteine
TRG: Telomere-related gene
FVC: Forced vital capacity
PPF: Progressive pulmonary fibrosis
PH: Pulmonary hypertension
MDT: Multidisciplinary team
GERD: Gastro-esophageal reflux disease
AI: Artificial intelligence
IT: Information technology
NSIP: Nonspecific interstitial pneumonia
MDD: Multi-disciplinary discussion
OSA: Obstructive sleep apnea
ILAs: Interstitial lung abnormalities

## References

[1] Travis WD, Costabel U, Hansell DM (2013). An official American Thoracic Society/European Respiratory Society statement: update of the international multidisciplinary classification of the idiopathic interstitial pneumonias. Am J Respir Crit Care Med.

[2] Bryan CS (1994). What is the Oslerian tradition?. Ann Intern Med.

[3] Vanfleteren LEGW, Kocks JWH, Stone IS (2014). Moving from the Oslerian paradigm to the post-genomic era: are asthma and COPD outdated terms?. Thorax.

[4] Woodruff PG, Agusti A, Roche N (2015). Current concepts in targeting chronic obstructive pulmonary disease pharmacotherapy: making progress towards personalised management. Lancet.

[5] Loscalzo J, Kohane I, Barabasi AL (2007). Human disease classification in the postgenomic era: a complex systems approach to human pathobiology. Mol Syst Biol.

[6] Bateman ED, Reddel HK, van Zyl-Smit RN (2015). The asthma–COPD overlap syndrome: a staging post to a revised taxonomy of chronic airways diseases?. Lancet Respir Med.

[7] Spagnolo P, Grunewald J, du Bois RM (2014). Genetic determinants of pulmonary fibrosis: evolving concepts. Lancet Respir Med.

[8] Kokosi MA, Margaritopoulos GA, Wells AU (2018). Personalised medicine in interstitial lung diseases. Eur Respir Rev.

[9] De Sadeleer LJ, Verleden SE, Schupp JC (2022). BAL transcriptomes characterize idiopathic pulmonary fibrosis endotypes with prognostic impact. Chest.

[10] Adegunsoye A, Morisset J, Newton CA (2021). Leukocyte telomere length and mycophenolate therapy in chronic hypersensitivity pneumonitis. Eur Respir J..

[11] McDonald VM, Fingleton J, Agusti A (2019). Treatable Traits: a new paradigm for 21st century management of chronic airway diseases. Eur Respir J.

[12] Agusti A, Bel E, Thomas M (2016). Treatable traits: toward precision medicine of chronic airway diseases. Eur Respir J.

[13] Boaventura R, Sibila O, Agusti A (2018). Treatable traits in bronchiectasis. Eur Respir J.

[14] Amati F, Spagnolo P, Oldham JM (2023). Treatable traits in interstitial lung diseases: a call to action. Lancet Respir Med.

[15] Hoffmann-Vold AM, Weigt SS, Saggar R (2019). Endotype-phenotyping may predict a treatment response in progressive fibrosing interstitial lung disease. EBioMedicine.

[16] Amati F, Bongiovanni G, Tonutti A (2023). Treatable traits in systemic sclerosis. Clin Rev Allergy Immunol.

[17] Adegunsoye A, Oldham JM, Chung JH (2018). Phenotypic clusters predict outcomes in a longitudinal interstitial lung disease cohort. Chest.

[18] Hamman L, Rich A (1944). Acute diffuse interstitial fibrosis of the lungs. Bull Johns Hopkins Hosp.

[19] Rubin E, Lubliner H (1957). The Hamman-Rich syndrome: review of the literature and analysis of 15 cases. Medicine (Baltimore).

[20] Sheridan LA, Harrison JH, Divertie MB (1964). The current status of idiopathic pulmonary fibrosis (Hamman-Rich Syndrome). Med Clin N Am.

[21] Gaensler EA, Moister VB, Hamm J (1964). Open-lung biopsy in diffuse pulmonary disease. N Engl J Med.

[22] Liebow A (1975). Definition and classification of interstitial pneumonias in human pathology. Prog Respir Dis.

[23] Todo G, Itoh H, Nakano Y, Dodo Y, Maeda H, Murata K, Odori T, Torizuka K, Izumi T, Oshima S (1982). High-resolution CT (HR-CT) for the evaluation of pulmonary peripheral disorders. Jpn J Clin Imaging.

[24] ATS/ERS. International multidisciplinary consensus classification of the idiopathic interstitial pneumonias. Am J Respir Crit Care Med 2002;165:277–304. 10.1164/ajrccm.165.2.ats01 11790668

[25] Richeldi L, du Bois RM, Raghu G (2014). Efficacy and safety of nintedanib in idiopathic pulmonary fibrosis. N Engl J Med.

[26] Richeldi L, Costabel U, Selman M (2011). Efficacy of a tyrosine kinase inhibitor in idiopathic pulmonary fibrosis. N Engl J Med.

[27] Raghu G, Brown KK, Bradford WZ (2004). A placebo-controlled trial of interferon gamma-1b in patients with idiopathic pulmonary fibrosis. N Engl J Med.

[28] Tashkin DP, Roth MD, Clements PJ (2016). Mycophenolate mofetil versus oral cyclophosphamide in scleroderma-related interstitial lung disease (SLS II): a randomised controlled, double-blind, parallel group trial. Lancet Respir Med.

[29] Idiopathic Pulmonary Fibrosis Clinical Research Network, Raghu G, Anstrom KJ, King TE Jr, Lasky JA, Martinez FJ. Prednisone, azathioprine, and N-acetylcysteine for pulmonary fibrosis. N Engl J Med. 2012;366(21):1968–1977. 10.1056/NEJMoa1113354 10.1056/NEJMoa1113354 PMC3422642 22607134

[30] Courtwright AM, El-Chemaly S (2019). Telomeres in Interstitial Lung Disease: The Short and the Long of It. Ann Am Thorac Soc.

[31] Oldham JM, Ma SF, Martinez FJ (2015). TOLLIP, MUC5B, and the response to N-acetylcysteine among individuals with idiopathic pulmonary fibrosis. Am J Respir Crit Care Med.

[32] Justet A, Thabut G, Manali E (2018). Safety and efficacy of pirfenidone in patients carrying telomerase complex mutation. Eur Respir J..

[33] Dressen A, Abbas AR, Cabanski C (2018). Analysis of protein-altering variants in telomerase genes and their association with MUC5B common variant status in patients with idiopathic pulmonary fibrosis: a candidate gene sequencing study. Lancet Respir Med.

[34] Newton CA, Zhang D, Oldham JM (2019). Telomere length and use of immunosuppressive medications in idiopathic pulmonary fibrosis. Am J Respir Crit Care Med.

[35] Schwarzkopf L, Witt S, Waelscher J, Polke M, Kreuter M (2018). Associations between comorbidities, their treatment and survival in patients with interstitial lung diseases—a claims data analysis. Respir Res.

[36] Kreuter M, Ehlers-Tenenbaum S, Palmowski K (2016). Impact of comorbidities on mortality in patients with idiopathic pulmonary fibrosis. PLoS ONE.

[37] Behr J, Kreuter M, Hoeper MM (2015). Management of patients with idiopathic pulmonary fibrosis in clinical practice: the INSIGHTS-IPF registry. Eur Respir J.

[38] Lee CT, Adegunsoye A, Chung JH (2021). Characteristics and prevalence of domestic and occupational inhalational exposures across interstitial lung diseases. Chest.

[39] Collins FS, Varmus H (2015). A new initiative on precision medicine. N Engl J Med.

[40] Jameson JL, Longo DL (2015). Precision medicine—personalized, problematic, and promising. N Engl J Med.

[41] Lotvall J, Akdis CA, Bacharier LB (2011). Asthma endotypes: a new approach to classification of disease entities within the asthma syndrome. J Allergy Clin Immunol.

[42] Agusti A (2014). The path to personalized medicine in COPD. Thorax.

[43] Ray A, Oriss TB, Wenzel SE (2015). Emerging molecular phenotypes of asthma. Am J Physiol Lung Cell Mol Physiol.

[44] McDonald VM, Clark VL, Cordova-Rivera L (2020). Targeting treatable traits in severe asthma: a randomised controlled trial. Eur Respir J.

[45] McDonald VM, Higgins I, Wood LG, Gibson PG (2013). Multidimensional assessment and tailored interventions for COPD: respiratory utopia or common sense?. Thorax.

[46] Zamora-Legoff JA, Krause ML, Crowson CS, Ryu JH, Matteson EL (2017). Progressive decline of lung function in rheumatoid arthritis-associated interstitial lung disease. Arthritis Rheumatol.

[47] Reiseter S, Gunnarsson R, Mogens Aalokken T, Lund MB, Mynarek G, Corander J (2018). Progression and mortality of interstitial lung disease in mixed connective tissue disease: a long-term observational nationwide cohort study. Rheumatology (Oxford).

[48] Olson AL, Gifford AH, Inase N, Fernández Pérez ER, Suda T (2018). The epidemiology of idiopathic pulmonary fibrosis and interstitial lung diseases at risk of a progressive-fibrosing phenotype. Eur Respir Rev.

[49] George PM, Spagnolo P, Kreuter M (2020). Progressive fibrosing interstitial lung disease: clinical uncertainties, consensus recommendations, and research priorities. Lancet Respir Med.

[50] Cottin V, Wollin L, Fischer A, Quaresma M, Stowasser S, Harari S (2019). Fibrosing interstitial lung diseases: knowns and unknowns. Eur Respir Rev..

[51] Raghu G, Remy-Jardin M, Richeldi L, et al. Idiopathic pulmonary fibrosis (an update) and progressive pulmonary fibrosis in adults: an official ATS/ERS/JRS/ALAT Clinical Practice Guideline. Am J Respir Crit Care Med. 2022;205(9). 10.1164/rccm.202202-0399ST PMC9851481 35486072

[52] Flaherty KR, Wells AU, Cottin V (2019). Nintedanib in progressive fibrosing interstitial lung diseases. N Engl J Med.

[53] Distler O, Highland KB, Gahlemann M (2019). Nintedanib for systemic sclerosis-associated interstitial lung disease. N Engl J Med.

[54] Wells AU, Flaherty KR, Brown KK (2020). Nintedanib in patients with progressive fibrosing interstitial lung diseases-subgroup analyses by interstitial lung disease diagnosis in the INBUILD trial: a randomised, double-blind, placebo-controlled, parallel-group trial. Lancet Respir Med.

[55] Amati F, Stainer A, Polelli V (2023). Efficacy of pirfenidone and nintedanib in interstitial lung diseases other than idiopathic pulmonary fibrosis: a systematic review. Int J Mol Sci.

[56] Maher TM, Corte TJ, Fischer A (2020). Pirfenidone in patients with unclassifiable progressive fibrosing interstitial lung disease: a double-blind, randomised, placebo-controlled, phase 2 trial. Lancet Respir Med.

[57] Behr J, Prasse A, Kreuter M (2021). Pirfenidone in patients with progressive fibrotic interstitial lung diseases other than idiopathic pulmonary fibrosis (RELIEF): a double-blind, randomised, placebo-controlled, phase 2b trial. Lancet Respir Med.

[58] Fernandez Perez ER, Swigris JJ, Forssen AV (2013). Identifying an inciting antigen is associated with improved survival in patients with chronic hypersensitivity pneumonitis. Chest.

[59] Salisbury ML, Myers JL, Belloli EA, Kazerooni EA, Martinez FJ, Flaherty KR (2017). Diagnosis and treatment of fibrotic hypersensitivity pneumonia. Where we stand and where we need to go. Am J Respir Crit Care Med.

[60] King CS, Nathan SD (2017). Idiopathic pulmonary fibrosis: effects and optimal management of comorbidities. Lancet Respir Med.

[61] Torrisi SE, Ley B, Kreuter M (2019). The added value of comorbidities in predicting survival in idiopathic pulmonary fibrosis: a multicentre observational study. Eur Respir J..

[62] Karampitsakos T, Spagnolo P, Mogulkoc N (2023). Lung cancer in patients with idiopathic pulmonary fibrosis: a retrospective multicentre study in Europe. Respirology.

[63] Raghu G, Amatto VC, Behr J (2015). Comorbidities in idiopathic pulmonary fibrosis patients: a systematic literature review. Eur Respir J.

[64] Dowman LM, McDonald CF, Hill CJ (2017). The evidence of benefits of exercise training in interstitial lung disease: a randomised controlled trial. Thorax.

[65] Nishiyama O, Yamazaki R, Sano H (2017). Fat-free mass index predicts survival in patients with idiopathic pulmonary fibrosis. Respirology.

[66] Pugashetti J, Graham J, Boctor N (2018). Weight loss as a predictor of mortality in patients with interstitial lung disease. Eur Respir J..

[67] Faverio P, Fumagalli A, Conti S (2022). Sarcopenia in idiopathic pulmonary fibrosis: a prospective study exploring prevalence, associated factors and diagnostic approach. Respir Res.

[68] Jouneau S, Lederlin M, Vernhet L, Thibault R (2019). Malnutrition in idiopathic pulmonary fibrosis: the great forgotten comorbidity!. Eur Respir J..

[69] Weill D, Benden C, Corris PA (2015). A consensus document for the selection of lung transplant candidates: 2014—an update from the Pulmonary Transplantation Council of the International Society for Heart and Lung Transplantation. J Heart Lung Transplant.

[70] Montgomery E, Macdonald PS, Newton PJ (2020). Frailty as a predictor of mortality in patients with interstitial lung disease referred for lung transplantation. Transplantation.

[71] Milne KM, Kwan JM, Guler S (2017). Frailty is common and strongly associated with dyspnoea severity in fibrotic interstitial lung disease. Respirology.

[72] Farooqi MAM, O'Hoski S, Goodwin S (2021). Prevalence and prognostic impact of physical frailty in interstitial lung disease: a prospective cohort. Respirology.

[73] Guler SA, Hur SA, Stickland MK (2022). Survival after inpatient or outpatient pulmonary rehabilitation in patients with fibrotic interstitial lung disease: a multicentre retrospective cohort study. Thorax.

[74] King CS, Shlobin OA (2020). The trouble with group 3 pulmonary hypertension in interstitial lung disease: dilemmas in diagnosis and the conundrum of treatment. Chest.

[75] Galiè N, Humbert M, Vachiery JL (2016). 2015 ESC/ERS Guidelines for the diagnosis and treatment of pulmonary hypertension: The Joint Task Force for the Diagnosis and Treatment of Pulmonary Hypertension of the European Society of Cardiology (ESC) and the European Respiratory Society (ERS): Endorsed by: Association for European Paediatric and Congenital Cardiology (AEPC), International Society for Heart and Lung Transplantation (ISHLT). Eur Heart J.

[76] Waxman A, Restrepo-Jaramillo R, Thenappan T (2021). Inhaled treprostinil in pulmonary hypertension due to interstitial lung disease. N Engl J Med.

[77] Winterbottom CJ, Shah RJ, Patterson KC (2018). Exposure to ambient particulate matter is associated with accelerated functional decline in idiopathic pulmonary fibrosis. Chest.

[78] Singh S, Collins BF, Bairwa M (2019). Hypersensitivity pneumonitis and its correlation with ambient air pollution in urban India. Eur Respir J.

[79] Singh S, Collins BF, Sharma BB (2017). Interstitial lung disease in India. Results of a prospective registry. Am J Respir Crit Care Med.

[80] Rice MB, Li W, Schwartz J (2019). Ambient air pollution exposure and risk and progression of interstitial lung abnormalities: the Framingham Heart Study. Thorax.

[81] Johannson KA, Vittinghoff E, Lee K (2014). Acute exacerbation of idiopathic pulmonary fibrosis associated with air pollution exposure. Eur Respir J.

[82] De Sadeleer LJ, Meert C, Yserbyt J (2018). Diagnostic ability of a dynamic multidisciplinary discussion in interstitial lung diseases: a retrospective observational study of 938 cases. Chest.

[83] Jo HE, Glaspole IN, Levin KC (2016). Clinical impact of the interstitial lung disease multidisciplinary service. Respirology.

[84] Grewal JS, Morisset J, Fisher JH (2019). Role of a regional multidisciplinary conference in the diagnosis of interstitial lung disease. Ann Am Thorac Soc.

[85] Tobin RW, Pope Ii CE, Pellegrini CA (1998). Increased prevalence of gastroesophageal reflux in patients with idiopathic pulmonary fibrosis. Am J Respir Crit Care Med.

[86] Soares RV, Forsythe A, Hogarth K (2011). Interstitial lung disease and gastroesophageal reflux disease: key role of esophageal function tests in the diagnosis and treatment. Arq Gastroenterol.

[87] Wang BR, Edwards R, Freiheit EA, Ma Y (2020). The pulmonary fibrosis foundation patient registry. Rationale, design, and methods. Ann Am Thorac Soc..

[88] Jo Helen E, Ian G, Christopher G (2017). Baseline characteristics of idiopathic pulmonary fibrosis: analysis from the Australian Idiopathic Pulmonary Fibrosis Registry. Eur Respir J.

[89] Farrand E, Anstrom KJ, Bernard G, Butte AJ, Iribarren C, Ley B (2019). Closing the evidence gap in interstitial lung disease. The promise of real-world data. Am J Respir Crit Care Med.

[90] Kuruvilla ME, Lee FE, Lee GB (2019). Understanding asthma phenotypes, endotypes, and mechanisms of disease. Clin Rev Allergy Immunol.

[91] Green RH, Brightling CE, McKenna S (2002). Asthma exacerbations and sputum eosinophil counts: a randomised controlled trial. Lancet.

[92] Papi A, Kostikas K, Wedzicha JA (2018). Dual bronchodilation response by exacerbation history and eosinophilia in the FLAME Study. Am J Respir Crit Care Med.

[93] Spagnolo P, Kropski JA, Jones MG (2021). Idiopathic pulmonary fibrosis: disease mechanisms and drug development. Pharmacol Ther.

[94] Brown AW, Shlobin OA, Weir N (2012). Dynamic patient counseling: a novel concept in idiopathic pulmonary fibrosis. Chest.

[95] Kim EJ, Collard HR, King TE (2009). Rheumatoid arthritis-associated interstitial lung disease: the relevance of histopathologic and radiographic pattern. Chest.

[96] Walsh SL, Sverzellati N, Deveraj A (2014). Connective tissue disease related fibrotic lung disease: high resolution computer tomographic and pulmonary function indices as prognostic determinants. Thorax.

[97] Peljto AL, Zhang Y, Fingerlin TE (2013). Association between the MUC5B promoter polymorphism and survival in patients with idiopathic pulmonary fibrosis. JAMA.

[98] Hunninghake GM, Hatabu H, Okajima Y (2013). MUC5B promoter polymorphism and interstitial lung abnormalities. N Engl J Med.

[99] Fingerlin TE, Murphy E, Zhang W (2013). Genome-wide association study identifies multiple susceptibility loci for pulmonary fibrosis. Nat Genet.

[100] Planas-Cerezales L, Arias-Salgado EG, Berastegui C (2021). Lung transplant improves survival and quality of life regardless of telomere dysfunction. Front Med (Lausanne)..

[101] Moore C, Blumhagen RZ, Yang IV (2019). Resequencing study confirms that host defense and cell senescence gene variants contribute to the risk of idiopathic pulmonary fibrosis. Am J Respir Crit Care Med.

[102] Noth I, Zhang Y, Ma SF (2013). Genetic variants associated with idiopathic pulmonary fibrosis susceptibility and mortality: a genome-wide association study. Lancet Respir Med.

[103] Juge PA, Lee JS, Ebstein E (2018). MUC5B promoter variant and rheumatoid arthritis with interstitial lung disease. N Engl J Med.

[104] Bonella F, Costabel U (2014). Biomarkers in connective tissue disease-associated interstitial lung disease. Semin Respir Crit Care Med.

[105] Tzouvelekis A, Herazo-Maya JD, Slade M (2017). Validation of the prognostic value of MMP-7 in idiopathic pulmonary fibrosis. Respirology.

[106] Guiot J, Henket M, Corhay JL, Moermans C, Louis R (2017). Sputum biomarkers in IPF: evidence for raised gene expression and protein level of IGFBP-2, IL-8 and MMP-7. PLoS ONE.

[107] Janssen R, Sato H, Grutters JC (2003). Study of Clara cell 16, KL-6, and surfactant protein-D in serum as disease markers in pulmonary sarcoidosis. Chest.

[108] Yamakawa H, Hagiwara E, Ikeda S (2019). Evaluation of changes in the serum levels of Krebs von den Lungen-6 and surfactant protein-D over time as important biomarkers in idiopathic fibrotic nonspecific interstitial pneumonia. Respir Investig..

[109] Okamoto T, Fujii M, Furusawa H, Tsuchiya K, Miyazaki Y, Inase N (2015). The usefulness of KL-6 and SP-D for the diagnosis and management of chronic hypersensitivity pneumonitis. Respir Med.

[110] Suga M, Iyonaga K, Okamoto T (2000). Characteristic elevation of matrix metalloproteinase activity in idiopathic interstitial pneumonias. Am J Respir Crit Care Med.

[111] Boot RG, Hollak CE, Verhoek M, Alberts C, Jonkers RE, Aerts JM (2010). Plasma chitotriosidase and CCL18 as surrogate markers for granulomatous macrophages in sarcoidosis. Clin Chim Acta.

[112] Lee JS, Lee EY, Ha YJ, Kang EH, Lee YJ, Song YW (2019). Serum KL-6 levels reflect the severity of interstitial lung disease associated with connective tissue disease. Arthritis Res Ther.

[113] Hamai K, Iwamoto H, Ishikawa N (2016). Comparative study of circulating MMP-7, CCL18, KL-6, SP-A, and SP-D as disease markers of idiopathic pulmonary fibrosis. Dis Markers.

[114] Song JW, Do KH, Jang SJ, Colby TV, Han S, Kim DS (2013). Blood biomarkers MMP-7 and SP-A: predictors of outcome in idiopathic pulmonary fibrosis. Chest.

[115] Korthagen NM, van Moorsel CH, Barlo NP (2011). Serum and BALF YKL-40 levels are predictors of survival in idiopathic pulmonary fibrosis. Respir Med.

[116] Prasse A, Probst C, Bargagli E (2009). Serum CC-chemokine ligand 18 concentration predicts outcome in idiopathic pulmonary fibrosis. Am J Respir Crit Care Med.

[117] De Lauretis A, Sestini P, Pantelidis P (2013). Serum interleukin 6 is predictive of early functional decline and mortality in interstitial lung disease associated with systemic sclerosis. J Rheumatol.

[118] Kuwana M, Shirai Y, Takeuchi T (2016). Elevated serum Krebs von den Lungen-6 in early disease predicts subsequent deterioration of pulmonary function in patients with systemic sclerosis and interstitial lung disease. J Rheumatol.

[119] Long X, He X, Ohshimo S (2017). Serum YKL-40 as predictor of outcome in hypersensitivity pneumonitis. Eur Respir J.

[120] Hoffmann-Vold AM, Tennøe AH, Garen T (2016). High level of chemokine CCL18 is associated with pulmonary function deterioration, lung fibrosis progression, and reduced survival in systemic sclerosis. Chest.

[121] Kubo S, Siebuhr AS, Bay-Jensen AC (2020). Correlation between serological biomarkers of extracellular matrix turnover and lung fibrosis and pulmonary artery hypertension in patients with systemic sclerosis. Int J Rheum Dis.

[122] Jessen H, Hoyer N, Prior TS (2021). Turnover of type I and III collagen predicts progression of idiopathic pulmonary fibrosis. Respir Res.

[123] Pardo A, Cabrera S, Maldonado M, Selman M (2016). Role of matrix metalloproteinases in the pathogenesis of idiopathic pulmonary fibrosis. Respir Res.

[124] O'Dwyer DN, Moore BB (2017). The role of periostin in lung fibrosis and airway remodeling. Cell Mol Life Sci.

[125] Agassandian M, Tedrow JR, Sembrat J (2015). VCAM-1 is a TGF-β1 inducible gene upregulated in idiopathic pulmonary fibrosis. Cell Signal.

[126] Alqalyoobi S, Adegunsoye A, Linderholm A (2020). Circulating plasma biomarkers of progressive interstitial lung disease. Am J Respir Crit Care Med.

[127] Vuga LJ, Tedrow JR, Pandit KV (2014). C-X-C motif chemokine 13 (CXCL13) is a prognostic biomarker of idiopathic pulmonary fibrosis. Am J Respir Crit Care Med.

[128] Campo I, Zorzetto M, Bonella F (2015). Facts and promises on lung biomarkers in interstitial lung diseases. Expert Rev Respir Med.

[129] Bowman WS, Newton CA, Linderholm AL (2022). Proteomic biomarkers of progressive fibrosing interstitial lung disease: a multicentre cohort analysis. Lancet Respir Med.

[130] Richeldi L, Scholand MB, Lynch DA (2021). Utility of a molecular classifier as a complement to high-resolution computed tomography to identify usual interstitial pneumonia. Am J Respir Crit Care Med.

[131] Walsh SLF, Humphries SM, Wells AU, Brown KK (2020). Imaging research in fibrotic lung disease; applying deep learning to unsolved problems. Lancet Respir Med.

[132] Bak SH, Park HY, Nam JH (2019). Predicting clinical outcome with phenotypic clusters using quantitative CT fibrosis and emphysema features in patients with idiopathic pulmonary fibrosis [published correction appears in PLoS One. 2019 Jun 5;14(6):e0218223]. PLoS ONE.

[133] Jacob J, Bartholmai BJ, Rajagopalan S (2017). Mortality prediction in idiopathic pulmonary fibrosis: evaluation of computer-based CT analysis with conventional severity measures. Eur Respir J..

[134] Walsh SLF, Calandriello L, Silva M, Sverzellati N (2018). Deep learning for classifying fibrotic lung disease on high-resolution computed tomography: a case-cohort study. Lancet Respir Med.

[135] Pankratz DG, Choi Y, Imtiaz U (2017). Usual interstitial pneumonia can be detected in transbronchial biopsies using machine learning. Ann Am Thorac Soc.

[136] Raghu G, Flaherty KR, Lederer DJ (2019). Use of a molecular classifier to identify usual interstitial pneumonia in conventional transbronchial lung biopsy samples: a prospective validation study. Lancet Respir Med.

[137] McDonald VM, Higgins I, Wood LG, Gibson PG (2013). Multidimensional assessment and tailored interventions for COPD: respiratory utopia or common sense?. Thorax.

[138] Park IN, Jegal Y, Kim DS (2009). Clinical course and lung function change of idiopathic nonspecific interstitial pneumonia. Eur Respir J.

[139] Romagnoli M, Nannini C, Piciucchi S (2011). Idiopathic nonspecific interstitial pneumonia: an interstitial lung disease associated with autoimmune disorders?. Eur Respir J.

[140] Bendstrup E, Hyldgaard C, Altraja A (2015). Organisation of diagnosis and treatment of idiopathic pulmonary fibrosis and other interstitial lung diseases in the Nordic countries. Eur Clin Respir J.

[141] Prades J, Remue E, van Hoof E (2015). Is it worth reorganising cancer services on the basis of multidisciplinary teams (MDTs)? A systematic review of the objectives and organisation of MDTs and their impact on patient outcomes. Health Policy.

[142] King TE, Bradford WZ, Castro-Bernardini S (2014). A phase 3 trial of pirfenidone in patients with idiopathic pulmonary fibrosis. N Engl J Med.

[143] Noble PW, Albera C, Bradford WZ (2011). Pirfenidone in patients with idiopathic pulmonary fibrosis (CAPACITY): two randomised trials. Lancet.

[144] Ryerson CJ, Urbania TH, Richeldi L (2013). Prevalence and prognosis of unclassifiable interstitial lung disease. Eur Respir J.

[145] Kim SY, Diggans J, Pankratz D, Huang J, Pagan M, Sindy N (2015). Classification of usual interstitial pneumonia in patients with interstitial lung disease: assessment of a machine learning approach using high-dimensional transcriptional data. Lancet Respir Med.

[146] Noell G, Faner R, Agustí A (2018). From systems biology to P4 medicine: applications in respiratory medicine. Eur Respir Rev.

[147] Flores M, Glusman G, Brogaard K, Price ND, Hood L (2013). P4 medicine: how systems medicine will transform the healthcare sector and society. Per Med.

[148] Hatabu H, Hunninghake GM, Lynch DA (2019). Interstitial lung abnormality: recognition and perspectives. Radiology.

[149] Hoyer N, Wille MMW, Thomsen LH (2018). Interstitial lung abnormalities are associated with increased mortality in smokers. Respir Med.

[150] Putman RK, Hatabu H, Araki T (2016). Association between interstitial lung abnormalities and all-cause mortality. JAMA.

